# The PKA-CREB system encoded by the honeybee genome

**DOI:** 10.1111/j.1365-2583.2006.00668.x

**Published:** 2006-10

**Authors:** D Eisenhardt, C Kühn, G Leboulle

**Keywords:** honeybee genome, CREB, PKA, bZIP, splicing, uORF, destabilizing elements

## Abstract

The cAMP-dependent kinase (PKA) plays a crucial part in long-term memory formation in the honeybee (*Apis mellifera*). One of the putative substrates of the PKA activity is the cAMP response element binding protein (CREB), a transcription factor in the bZIP protein family. We searched the honeybee genome to characterize genes from the CREB/CREM and the PKA families. We identified two genes that encode regulatory subunits and three genes encode catalytic subunits of PKA. Eight genes code for bZIP proteins, but only one gene was found that encodes a member of the CREB/CREM family. The phylogenetic relationship of these genes was analysed with their *Drosophila* and human counterparts.

## Introduction

The honeybee (*Apis mellifera*) is a model organism for studying learning and memory formation and its underlying mechanisms (Menzel, 2001). The cAMP-dependent kinase (PKA) plays a crucial part in a multitude of cellular processes and in the formation of long-term memories in the honeybee (Fiala *et al*., 1999; Friedrich *et al*., 2004; Taylor *et al*., 2005). The PKA holoenzyme is an inactive tetramer composed of two regulatory (R) and two catalytic (C) subunits. Binding of two cAMP molecules to the R subunit releases the catalytic subunit, which can phosphorylate specific substrates, such as cAMP response element binding protein (CREB) (Tasken & Aandahl, 2004). PKA phosphorylates CREB within a kinase-inducible domain (KID). This results in the expression of CREB target genes that are thought to contribute to the formation of long-term synaptic plasticity underlying long-term memory consolidation (Kandel, 2001; Lonze & Ginty, 2002).

CREB belongs to the CREB/CREM subgroup of transcription factors, which, in turn, is a member of the super-family of basic region-leucin zipper (bZIP) proteins (Hai & Hartman, 2001). In mammals, this subgroup includes CREB, CREM and ATF-1, which are encoded by three genes (Hai & Hartmann, 2001). In the honeybee, eight CREB variants that derive from one gene have been cloned (Eisenhardt *et al*., 2003).

The subunits of PKA are encoded by several genes, each giving rise to a variety of splice variants. In mammals, four regulatory subunits are encoded by four genes (*prkar-1α*, *-1β* and *prkar-2α* and *-2β*) and three genes encode the catalytic subunits (*prkac-α*, *-β* and *-γ*), referred to here as the ‘classical’ catalytic subunits (Francis & Corbin, 1999). Another family of catalytic subunits has been described. It comprises four members, among them *prkx*, whose product associates with the R1 subunit and is implicated in cellular morphogenesis (Schiebel *et al*., 1997; Zimmermann *et al*., 1999; Li *et al*., 2002). Other genes in this family are *prky* and *ENSG00000177648*, a predicted gene from the human genome. In *Drosophila*, two genes, *Pka-R1* and *Pka-R2* (also known as *DR1* and *DR2*), encode the regulatory subunits. Three genes, *Pka-C1*, *-C2* and *-C3* (also known as *DC0*, *DC1* and *DC2*) encode the catalytic subunits (Kalderon & Rubin, 1988). In the honeybee one catalytic subunit (*AmCO*) and one regulatory subunit (*Am pka-r2*) of PKA, have been identified (Eisenhardt *et al*., 2001; Leboulle & Muller, 2004). To date it is unknown how many genes encode subunits of PKA and CREB proteins in the honeybee. This poses a problem in physiological studies, because it is always unclear how many proteins are targeted by pharmacological treatments or gene-specific down-regulation techniques (anti-sense oligos, RNAi).

Hence, in this study the analysis of the honeybee genome revealed two genes encoding regulatory subunits and three genes encoding catalytic subunits of PKA. Eight genes were identified that code for bZIP proteins, and only one belonged to the CREB/CREM subgroup of transcription factors. This gene, *Am CREB*, has already been identified in a previous experimental approach (Eisenhardt *et al*., 2003), but the honeybee genome enabled us to completely analyse its structure.

## Results and discussion

### The regulatory and catalytic subunits of PKA

#### Honeybee genes of the PKA family

The genome was searched to identify all the genes of the PKA family. We will refer to the *Drosophila* genes as *Dm Pka-R1*, *-R2*, *-C1*, *-C2* and *-C3*. The honeybee nomenclature will be based on the *Drosophila* one but will add the prefix Am to the name (e.g. *Am pka-c1* instead of *AmCO*).

The known PKA genes from the honeybee, *Drosophila* and humans were compared with the version 2.0 (v.2.0) assembly of the honeybee genome. The search for regulatory subunits led to the identification of two genes, *Am pka-r1* and *Am pka-r2*. The search for catalytic subunits identified three genes, *Am pka-c1*, *Am pka-c2* and *Am pka-c3*. Four of the identified PKA genes (*Am pka-r1*, *-r2*, *-c1*, and *-c3*) matched portions of the genome that were already identified as genes in the v.2.0 assembly (Table 1). Their official accession numbers were extracted from the official_gene_set_1_cds database that contains potential coding sequences (CDS) deduced from the honeybee genome (Table 1).

**Table 1:** Identified honeybee regulatory and catalytic subunits of PKA

Official Acc n˚*	Gene	Synonyms	Gene id†	Group‡	Coordinates (start/end)§	Contig name¶	mRNA Acc n˚**	Protein length
GB13272-RA	*Am pka-r1*		ENSAPMG00000009757	GroupUn−	14661479 14655314	Contig6578		372 aa
GB14637-RA	*Am pka-r2*		ENSAPMG00000011420	Group10+	4896980 4902889	Contig4009	AJ698737	383 aa
GB17175-RA	*Am pka-c1*	*AmCO*	ENSAPMG00000012575	Group2+	12221620 12222681	Contig1151	AJ271674	353 aa
GB16164-RA	*Am pka-c2*		–	Group1+	19416843 19417886	Contig613		348 aa
GB14368-RA	*Am pka-c3*		ENSAPMG00000007881	Group10−	6282978 6286741	Contig4055		331 aa

* Accession numbers as they were attributed in the official_gene_set_1_cds honeybee genome database.

† Accession numbers of the predicted genes in the v.2.0. assembly of the genome.

‡ Location of the genes within their respective groups and the DNA strand (+ or –) containing the gene.

§ Coordinates of the genes within their groups.

¶ Contigs containing the genes.

** GenBank/EMBL accession numbers.

#### Analysis of the regulatory subunits – phylogenetic analysis

**Figure 1:**
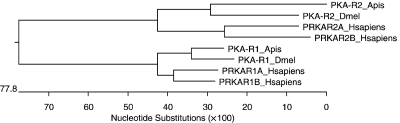
Phylogenetic tree of PKA regulatory subunits. An unrooted phylogenetic tree (cladogram) was generated from a ClustalW multiple alignment of honeybee (Apis) regulatory subunits with their *Drosophila* (Dmel) and human (Hsapiens) homologues. The branches are unbalanced to force branch distances to correspond to sequence divergence.

The deduced amino acid sequences of the honeybee regulatory subunits, Am PKA-R1 and Am PKA-R2 were aligned with their *Drosophila* and human homologues and a phylogenetic tree was generated (Fig. 1). R1 and R2 subunits were clustered in two separate groups. In each group, the insect and the human sequences formed separated subgroups. The highest degree of similarity was found between Am PKA-R1 and Dm PKA-R1 (82.8% identical amino acids). The degree of similarity between Am PKA-R1 and the human PKA-R1α and PKA-R1β was 74.8% and 72.7%, respectively. The degree of similarity between Am PKA-R2 and Dm PKA-R2 was 67%. The degree of similarity of Am PKA-R2 with PKA-R2α and PKA-R2β was 51.8% and 50.3%, respectively (Fig. 1). This supports previous assumptions, which proposed that the human subunits are the result of gene duplications that occurred after the separation of mammals and arthropods during evolution (Canaves & Taylor, 2002).

#### Analysis of the regulatory subunits – domain analysis of the regulatory subunits

On the basis of sequence alignment, the different domains constituting the regulatory subunits were identified and compared with their human and *Drosophila* counterparts. The R-subunits have a conserved structure with a dimerization/docking (D/D) domain at the N-terminus that provides a docking site for anchoring proteins. The D/D is joined to cAMP binding domains A and B by an extended linker comprising an auto-inhibitory sequence (Taylor *et al*., 2005).

All domains were highly conserved and of similar length between species. The Am PKA-R1 domains showed the highest degree of similarity with the corresponding domains of the *Drosophila* and human R1 subunits (Table 2). The same was true for the R2 subunits, although the similarity indexes were lower (Table 2). This, together with the observation of long branches in the R2 subgroup of the phylogenetic tree, suggests that these subunits evolved more rapidly than the R1 subunits. In all R subunits, the cAMP-binding domains A and B showed the highest similarity index compared with those of D/D domains, probably because the interaction of domains A and B with cAMP imposes constraints on the evolution of these domains.

**Table 2:** Comparison of the honeybee regulatory subunits domains

		Percent identity*					
		
		*Drosophila*†		Human†			
			
		Dm PKA-R1	Dm PKA-R2	PRKA-R1α	PRKA-R1β	PRKA-R2α	PRKA-R2β
		
	position‡	X16970	AF274008	NM_002734	NM_002735	NM_004157	NM_002736
**D/D domain**							
Am PKA-R2	1–47	14.9	**50**	10.6	10.6	**36.6**	**35**
Am PKA-R1	1–57	**75.9**	21.7	**66.7**	**61.1**	19.5	15
**Domain A**							
Am PKA-R2	105–253	46.5	**68.1**	48.6	47.2	**61.5**	**62.8**
Am PKA-R1	108–251	**90.3**	46.5	**83.3**	**82.6**	43.1	45.1
**Domain B**							
Am PKA-	254–370	35.9	**67.5**	36.8	39.3	**57.3**	**56.4**
Am PKA-	252–369	**89**	45.3	**83.1**	**83.9**	39.8	39

* The percentage of identical amino acids between the honeybee, the *Drosophila* and the human subunits with the highest similarity index, indicated in bold.

† The *Drosophila* and human subunits and their GenBank/EMBL accession numbers.

‡ Location in amino acids of the different honeybee domains within the protein.

#### Analysis of the regulatory subunits – differences between Am PKA-R1 and Am PKA-R2

One of the most important differences between R1 and R2, which is found in the honeybee and in all other species analysed so far, is the auto-inhibitory site. The latter is characterized by the consensus sequence R-R-x-[AG] in R1 and by the consensus sequence R-R-x-S in R2 subunits, where the serine residue is susceptible to phosphorylation.

Based on this sequence analysis, it was concluded that the *Am pka-r1* and *Am pka-r2* genes of the honeybee are orthologues of the regulatory subunits described in humans and *Drosophila*. Nevertheless, the R2 subunit of the honeybee can be synergistically activated by cAMP and cGMP, a property that was not observed in mammals (Leboulle & Muller, 2004).

#### Analysis of the catalytic subunits – phylogenetic analysis

**Figure 2:**
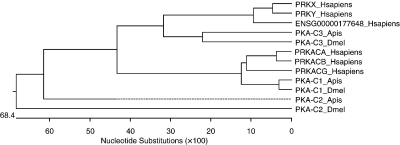
Phylogenetic tree of PKA catalytic subunits. An unrooted phylogenetic tree (phenogram) was generated from a ClustalW multiple alignment of honeybee (Apis) catalytic subunits with their *Drosophila* (Dmel) and human (Hsapiens) homologues. The branches are balanced to average the distances between ancestors in the tree. Dotted lines indicate a negative branch length.

The deduced amino acid sequences of the honeybee catalytic subunits, Am PKA-C1, Am PKA-C2 and Am PKA-C3, were aligned with their *Drosophila* and human homologues and a phylogenetic tree was generated (Fig. 2).

The first sequences diverging in the tree were Am PKA-C2 and Dm PKA-C2. They were located on branches generated from different nodes and shared 57.1% identical amino acids. These two insect subunits were not related to any human counterparts.

Am PKA-C3 clustered with Dm PKA-C3 (46.1% of identical amino acids), PRKX (70.6% of identical amino acids), PRKY (69.6% of identical amino acids) and the gene ENSG00000177648 (67.5% of identical amino acids). Interestingly, the highest similarity was found between the honeybee and the human proteins.

Am PKA-C1 subunits clustered with Dm PKA-C1 (96% of identical amino acids) and the ‘classical’ human catalytic subunits (PKA-Cα, PKA-Cβ and PKA-Cγ; 88.3%, 87.6% and 83.7% of identical amino acids). The clustering of the human subunits in subgroups separated from their insect homologues suggest that they are the result of gene duplications which occurred after mammals and arthropods diverged during evolution.

#### Analysis of the catalytic subunits – domain analysis of the catalytic subunits

The C-subunits are asymmetric bilobate molecules that exist in two conformational states described as open and closed. The catalytic site lies in a deep cleft bisecting the two lobes. The smaller lobe is associated primarily with Mg^2+^/ATP binding and the large lobe provides the interface for the interaction with protein/peptide substrates. The catalytic subunit contains extensions at the NH_2_-terminus and the C-terminus. Both segments interact with the lobes and are implicated in defining localization and protein/protein interactions (Taylor *et al*., 2005). The three honeybee subunits were characterized by conserved consensus sequences specific for the family of PKA catalytic subunits (Table S1 – see Supplementary material). These sequences constitute conserved loops that contribute directly to Mg^2+^/ATP binding or catalysis: a glycine-rich loop, located in the NH_2_-terminal portion of the smaller lobe, and two loops located in the large lobe (Knighton *et al*., 1991). In contrast, the N- and C-terminal domains were less conserved (Table 3): The N-terminal domains were about 40 amino acids long in all catalytic subunits, except in Am PKA-C3, which had a short domain (19 amino acids) and in Dm PKA-C3, with a very long one (188 amino acids). The C-terminal domains were about 50 amino acids long, except in PRKY, where it was absent.

**Table 3:** Comparison of the honeybee catalytic subunits domains with their *Drosophila* and human homologues

		Percent identity*								
		
		*Drosophila*†			Human†					
			
		Dm PKA-C1	Dm PKA-C2	Dm PKA-C3	PRKCα	PRKACβ	PRKACγ	PRKX	PRKY	ENSG00000177648
		
	position‡	X16969	X16960	X16961	NM_002730	NM_002731	NM_002732	P51817	O43930	
**N-term**										
Am PKA-C1	1–41	**82.9**	20	14.6	**51.3**	**46.2**	**41**	9.8	14.6	?
Am PKA-C2	1–36	19.4	**22.2**	8.3	**13.9**	**16.7**	**19.4**	5.6	8.3	?
Am PKA-C3	1–19	15.8	10.5	**31.6**	**21.1**	**21.1**	**21.1**	**21.1**	15.8	?
**small Lobe**										
Am PKA-C1	42–127	**93**	44.2	48.8	**82.6**	**81.4**	**75.6**	43	39.5	29.1
Am PKA-C2	37–122	**62.8**	43	41.9	**61.6**	**65.1**	**53.5**	38.4	34.9	25.6
Am PKA-C3	20–103	45.2	33.3	**52.4**	46.4	44	40.5	**50**	**47.6**	22.6
**large Lobe**										
Am PKA-C1	128–302	**97.7**	52	62.9	**90.9**	**90.3**	**82.9**	60	57.8	56.5
Am PKA-C2	123–297	**68.6**	52	58.3	**68**	**68**	**64**	53.1	52.4	47.2
Am PKA-C3	104–278	65.1	44.6	**82.9**	64	64	62.9	**76**	**72.1**	**71.4**
**C-term**										
Am PKA-C1	303–353	**92.2**	9.8	29.4	**76.5**	**76.5**	**76.5**	35.3	–	35.3
Am PKA-C2	298–348	**45.1**	5.9	29.4	**43.1**	**43.1**	**43.1**	25.5	–	25.5
Am PKA-C3	279–331	31.4	7.5	**41.5**	31.4	29.4	25.5	**47.2**	–	**45.3**

* The percentage of identical amino acids between the honeybee, the *Drosophila* and the human subunits with the highest similarity index indicated in bold.

† The *Drosophila* and human subunits and their GenBank/EMBL accession numbers.

‡ Location in amino acids of the different honeybee domains within the protein.

There was a close relationship between the different domains of Am PKA-C1 and those of Dm PKA-C1 and of the human family of ‘classical’ catalytic subunits (Table 3). Am PKA-C3 was closely related to Dm PKA-C3 and to the PRKX family of catalytic subunits, although to a lesser degree (Table 3). Am PKA-C2 is an atypical subunit. Its domains were more related to the respective domains of Dm PKA-C1 and of the human family of ‘classical’ catalytic subunits. Except for the N-terminal domain of Am PKA-C2, which was more related to the corresponding domain of Dm PKA-C2 (Table 3).

It is known that Am PKA-C1 is implicated in the formation of long-term memory (Fiala *et al*., 1999). The role of Am PKA-C2 and Am PKA-C3 is not known. PRKX, the homologue of Am PKA-C3 in mammals, is involved in cellular morphogenesis; mutant analysis in *Drosophila* showed that Dm PKA-C3 is not an essential gene (Melendez *et al*., 1995; Li *et al*., 2002).

#### Analysis of the genes structure

**Figure 3:**
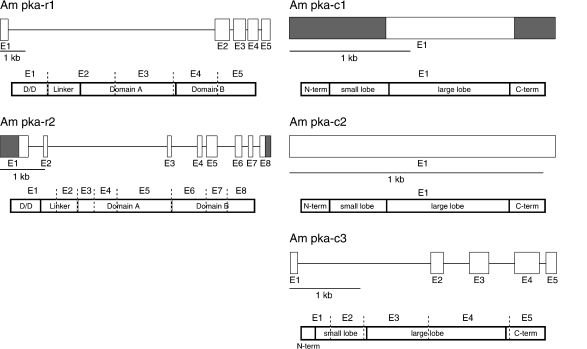
Structure of PKA genes. Above: Exons composing the genes are represented by boxes. White boxes: CDS; grey boxes: 5′- and 3′-UTRs. A line between boxes represents introns. Below: Putative protein sequences of the R and C subunits. The domains constituting the proteins are designated: D/D (dimerization/docking domain), domain A and B (cAMP-binding domains A and B), N-term and C-term (N-terminal and C-terminal domains), small and large lobes (small and large catalytic lobes). Dashed lines = borders of the exons.

*Am pka-r1* and *Am pka-c3* genes were composed of five exons covering the coding sequences (CDS); *Am pka-r2* was composed of eight exons (Fig. 3). Almost all of the genes’ splice sites were characterized by the GT-AG intron boundaries. A non-canonical GC-AG splice site was found in the intron 5 of *Am pka-r2* (Burset *et al*., 2000). The exon structure of the genes was often correlated with the putative structural and functional properties of the proteins. In mammals and in *Drosophila*, several splice variants were described for the catalytic and the regulatory subunits (Kalderon & Rubin, 1988; Dahle *et al*., 2001; Orstavik *et al*., 2001, 2005). These are characterized by a rearrangement of the untranslated regions (UTRs) or of the coding region. The structure of *Am pka-r1*, *Am pka-r2* and *Am pka-c3* suggest that some splice variants might be expressed. In contrast, *Am pka-c1* and *Am pkc-c2* most likely express only one isoform, because they are composed of a single exon. However, it cannot be excluded that additional non-coding exons are components of these genes and increase the diversity of variants expressed in the honeybee. This is supported by the fact that the 5′-UTR of *Am pka-c1* contains upstream open reading frames (uORFs) (Eisenhardt *et al*., 2001). uORFs have been shown to be involved in the regulation of translation efficiency and localization-dependent translation (Meijer & Thomas, 2002; Kozak, 2002). These regulatory regions were also found in *Drosophila* and murine catalytic subunits (Kalderon & Rubin, 1988; Guthrie *et al*., 1997) and might therefore indicate a conserved mechanism in the regulation of the expression of catalytic subunits.

### The cAMP response element binding protein

#### Identifying Apis mellifera members of the CREB/CREM family of transcription factors

In mammals three different genes encode CREB, CREM and ATF-1, all members of the CREB/CREM subgroup of the ATF/CREB family of transcription factors (Hai & Hartman, 2001). Here we wanted to know how many genes make up the CREB/CREM subgroup in the honeybee.

The proteins in the CREB/ATF family of transcription factors can be grouped into subgroups according to their amino acid similarity both inside and outside the bZIP domain (Hai & Hartman, 2001). Therefore we searched the official_gene_set_1_cds database for members of the CREB/CREM transcription factor-family using the bZIP motive of the previously identified honeybee *AmCREB* gene (Eisenhardt *et al*., 2003), the vertebrate CREB/CREM bZIP motive, as well as the vertebrate CREB and CREM proteins without the bZIP motive. When using the bZIP motive as a query sequence, we identified eight potential open reading frames (ORF) coding for proteins containing a bZIP domain as defined in the prosite database (PS50217 –Table 4). Only one ORF (GB11585) was retrieved when using the mammalian CREB and CREM proteins without their bZIP domain as the query sequence.

**Table 4:** Honeybee bZIP proteins and Drosophila and human counterparts

Official acc. no.*	Gene	Length/aa	bZIP domain position†	*Drosophila* (acc. no.) position: identity/homology‡	Human (acc. no.) position: identity/homology‡
GB 18117	AmBBF-2	424	352–415	BBF-2 (CAA45771) 25–132: 31%/46% 296–403: 77%/85%	CREB3L1 (AAH14097): 249–403: 50%/63%
GB 11585	AmCREB	305	247–298	dCREB-2 (NP_996507): 57–305: 34%/46%	CREB (CAA42620): 12–305: 37%/49%
GB 11753	ATF-3-like	269	131–194	A-A3 (NP_620473): 27–216: 36%/51%	ATF-3 (CAH72655): 126–199: 58%/81%
GB 12004	Jun-like	264	187–250	JUN (CAA73154): 60–257: 42%/62%	JUN-D (NP_005345): 25–258: 38%/51%
GB 16435	ATF-6-like	618	187–244	CG3136-PC (NP_995745): 147–533: 28%/41%	ATF-6 (NP_031374): 184–525: 32%/53%
GB 11712	CREB-H-like	600	343–406	BBF-2 (CAA45771): 291–414: 45%/60%	CREB-H (BAD38649): 313–418: 61%/77%
GB 18094	mafA-like	438	312–375	traffic jam (AAP88969): 266–373:64%/76%	MafA (AAL89527): 265–378: 52%/74%
GB 12212	Fos-like	300	201–264	dFRA (P21525): 183–283: 53%/74%	–

* Accession numbers as they were attributed in the official_gene_set_1_cds database of the honeybee genome.

† Location in amino acids of the bZIP domain as defined by prosite (PS50217) within the honeybee protein.

‡ Location of identity/homology with *Drosophila* and human homologues.

We subjected all identified sequences to a BLASTp-screen of general protein databases. This revealed that all of the identified honeybee sequences were homologous to already known bZIP-proteins in the region of the bZIP domain and in its proximity (Table 4). One of these sequences (*AmCREB*, GB11585) was identical with one of the previously identified splice variants of the *AmCREB* gene, *AmCREB-5* (Eisenhardt *et al*., 2003). The highest prediction values of the Blast search were found with a *Drosophila* dCREB-2 variant and the human CREB protein (Short *et al*., 1991; Yin *et al*., 1995). The remaining sequences encoded homologues of already-characterized *Drosophila* and human bZIP proteins (Table 4).

Genes encoding the identified bZIP proteins were retrieved by searching the v2.0 assembly of the honeybee genome. Five of the eight sequences were encoded by genomic sequences that had previously been predicted to be genes in the Ensembl database (v35) (Table 5). These genes were the *AmCREB*, *ATF-3-like*, *Jun-like*, *ATF-6-like*, *mafA-like* and *CREB-H like*. The *CREB-H* like gene spanned two formerly predicted genes (Table 5). In the previous annotation the two remaining genes, *AmBBF-2* and *FOS-like*, were not predicted to be genes or parts of genes (Table 5).

**Table 5:** Genomic location of honeybee bZIP genes

Official Acc no.*	Gene	Gene id†	Group‡	Coordinates (start/end)§	Contig name¶	Coordinates (start/end)**
GB18117	*AmBBF-2*	–	GroupUn (+)	272842 277092	Contig5940	17779 22029
GB11585	*AmCREB*	ENSAPMG00000005663	Group1 (+)	10958827 10966895	Contig392	2976 11044
GB11753	*ATF-3-like*	ENSAPMG00000007133	Group9 (+)	553499 555015	Contig3544	32020 33536
GB12004	*Jun-like*	ENSAPMG00000013115	Group9 (+)	1278335 1279129	Contig3576	4175 4969
GB12212	*Fos-like*	–	Group5 (+)	6319459 6348619	Contig2093 Contig2094	6818 6949 22507 23771
GB16435	*ATF-6-like*	ENSAPMG000000015281	GroupUn (+)	87526554 87530001	Contig9763 Contig9764	630 1650 817 1880
GB11712	*CREB-H-like*	ENSAPMG00000015253 ENSAPMG00000013733	Group14 (+)	3058861 3066597	Contig5254	1491 9227
GB18094	*mafA-like*	ENSAPMG00000009819	GroupUn (+)	9408488 9413178	Contig6339	11256 15946

* Accession numbers as they were attributed in the official_gene_set_1_cds honeybee genome database.

† Accession numbers of the predicted genes in the v.2.0. assembly of the genome.

‡ Location of the genes within their respective groups and the DNA strand (+ or –) containing the gene.

§ Coordinates of the genes within their groups.

¶ Contigs containing the genes.

** Location of the genes within their contig.

An analysis of a gene set of the honeybee genome (Zdobnov, 2005, http://cegg.lenige.ch/SUPPL/Bee/top500_domains.html) revealed 20 bZIP genes of the 10 157 annotated ones. Hence in the present analysis we did not identify all the honeybee genes that encode bZIP proteins. This is probably due to the fact that we screened the genome only for proteins having a bZIP domain closely related to the CREB bZIP domain.

#### Only one gene encodes a member of the CREB/CREM family of transcription factors

**Figure 4:**
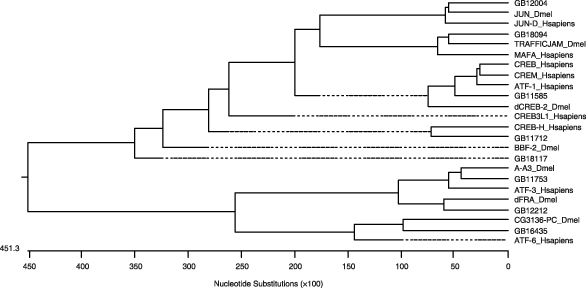
Phylogenetic tree of bZIP proteins. An unrooted phylogenetic tree (phenogram) was generated from a ClustalV multiple alignment of honeybee (Apis) bZIP proteins with their *Drosophila* (Dmel) and human (Hsapiens) homologues and human CREM and ATF-1. The branches are balanced to average the distances between ancestors in the tree. Dotted lines indicate a negative branch length.

A phylogenetic analysis was carried out, based on a ClustalV alignment. In this alignment the deduced amino acids sequences of the eight identified honeybee bZIP sequences, their human and *Drosophila* homologues, and the other two members of the human CREB/CREM subgroup, CREM and ATF-1 (accession numbers AAC60616 and AAB25878; Liu *et al.*, 1993; Masquilier *et al*., 1993), were used. Each honeybee sequence clustered with its *Drosophila* and human counterparts. The identified AmCREB sequence (GB11585) clustered with the *Drosophila* dCREB2 and with the human CREB, CREM and ATF-1 proteins, revealing their close relationship (Fig. 4). None of the other honeybee bZIP proteins was as closely related to this subgroup of transcription factors. We concluded from this screen that the *AmCREB* gene was the only member of the CREB/CREM family of transcription factors. Also in *Drosophila* only one member of the CREB/CREM family can be found. Fassler *et al*. (2002) analysed the *Drosophila* genome for bZIP proteins. They found 27 putative proteins with bZIP domains, one of which is a dCREB 2 protein. Only this protein clusters with a human CREM protein in a phylogenetic analysis. A second bZIP protein termed dCREBA (also known as BBF-2; Abel *et al.*, 1992; Smolik *et al*., 1992) is closely related to the human Oasis family but not to CREB or CREM (Fassler *et al*., 2002). Hence in the honeybee and in *Drosophila* the number of members of the CREB/CREM subgroup is reduced from three to one when compared with humans.

In the honeybee genome 0.2% of the genes contain a bZIP domain according to the Interpro database (IPR004827). This mirrors the fraction of bZIP proteins encoding genes in the genome of *Drosophila* (0.15%) and humans (0.22%) (Zdobnov, 2005; http://cegg.lenige.ch/SUPPL/Bee/top500_domains.html). Accordingly, a specific fraction of genes encodes bZIP proteins in every genome, rather than a certain number of genes. Hence the absolute number of genes encoding bZIP proteins in humans doubles the number in *Drosophila* and the honeybee (Poels & Vanden Broeck, 2004; Zdobnov, 2005; http://cegg.lenige.ch/SUPPL/Bee/top500_domains.html). In this light it is not surprising that the number of genes from the CREB/CREM family of transcription factors is higher in humans than in the honeybee and *Drosophila* (Fassler *et al*., 2002). Nevertheless, the functional relevance of this difference remains to be elucidated.

#### The structure of the AmCREB gene

**Figure 5:**
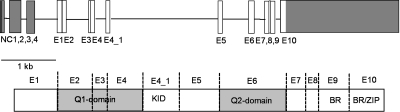
Structure of the AmCREB gene. Above: Exons composing the genes are represented by boxes; White boxes: CDS Exons; grey boxes 5′- and 3′-UTRs. A line between boxes represents introns. Below: A putative AmCREB peptide that consists of all CDS exons. Functionally relevant domains: Q1-domain (glutamine-rich domain), KID domain (kinase-inducible domain), Q2-domain (glutamine-rich domain), BR (basic region), ZIP (leucin zipper). Dashed lines = borders of the exons.

In a previous study, eight splice variants of the *AmCREB* gene have been isolated in a cDNA library screen (AmCREB-1–4) and in an RT–PCR experiment (AmCREB-5–8). AmCREB-1–4 contain coding and 5′- and -3′ non-coding regions, whereas AmCREB-5–8 contain only coding regions, due to the primers that were used (Eisenhardt *et al*., 2003). In this previous study the genomic structure of the *AmCREB* gene was experimentally explored, although not completely due to technical difficulties (Eisenhardt *et al*., 2003). Meanwhile the honeybee genome allowed us to analyse comprehensively the structure of this gene. We identified 15 exons and nine introns that were alternatively spliced (Table S2, Fig. 5). In comparison with a previous study (Eisenhardt *et al*., 2003), five additional exons (NC1–4 and E4_1) and four additional introns were identified (Table S2, Fig. 5). The introns were renamed I-1 to I-9. E10 was found to be much longer than previously described, because it coded for the huge 3′-non-coding region of the AmCREB transcripts (Table S2, Fig. 5).

#### Exons in the 5′-UTR of the AmCREB gene

We analysed the 5′-UTRs of AmCREB-1–4 (Eisenhardt *et al*., 2003). It turned out that these AmCREB cDNAs contained different 5′-UTRs that are composed of the four exons NC1–4 (Table S2, Fig. 5). Five of the exon–intron borders matched the canonical GT/AG splice sites (Akker *et al*., 2001), but non-canonical GT/AC splice sites were also found (Table S2). The exon NC1 and the intron I1 both terminated with the 3′-splice site AC instead of AG. We were not able to identify the corresponding 5′-splice sites. We suppose that they are part of the so-called AT-AC introns, which have AT and AC at their ends instead of GT and AG (Wu & Krainer, 1999).

The exons NC3 and NC4 contained uORFs (Geballe, 1996) that encode 2–46 amino acids. uORFs are involved in the regulation of translation (Meijer & Thomas, 2002; Kozak, 2002). Therefore, it might well be that uORFs in the 5′-UTR of AmCREB hint at a differential control of the translation of the specific AmCREB splice variants. uORFs are also located in the 5′-UTR of the *Drosophila* dCREB-2 (Usui *et al*., 1993). This underscores a possible functional relevance of the uORFs found in NC3 and NC4.

#### The coding region of the AmCREB gene

Our analyses revealed one additional exon (E4_1) and two introns (I5 and I6) in the coding region of the gene. The exon E4_1 was originally thought to be part of E4, but E4_1 is an extra exon, which was separated from the now-shorter E4 by the intron I5. The exon–intron borders of I5, E4_1 and I6 were characterized by the consensus GT/AG splice site (Akker *et al*., 2001 – Table S2).

The exon structure of the *AmCREB* gene correlated with the putative structural and functional properties of the encoded proteins (Fig. 5). This has been demonstrated for the mammalian members of the CREB/CREM family and for the *Drosophila dCREB-2* gene (Ruppert *et al*., 1992; Laoide *et al*., 1993, Yin *et al*., 1995). The identification of exon E4_1 fitted into this picture: the ‘new’ exon E4 together with E2 and E3 encoded the Q1 domain, which is important for the transcriptional activation of the CREB target genes (Gonzalez *et al*., 1991; Quinn, 1993). Exon E4_1 encoded the KID domain containing the PKA consensus sequences necessary for the activation CREB. The other functional domains were also encoded by one or more exons: the Q2 domain by E6 and the bZIP domain by E9 and E10 (Table S2, Fig. 5).

#### The 3′-UTR of the AmCREB gene

The 3′-UTRs of AmCREB-1–4 was part of exon E10. It encoded the 3′-end of the leucine zipper and contained the 3′-UTR stretching over at least 2418 nucleotides. The non-coding part of E10 was AT-rich (> 75%). It contained eight polyadenylation signals (PAS) (AATAAA or ATTAAA) (Wahle & Keller, 1992) and 13 copies of the ATTTA motive, which is one of the so-called AU-rich elements (AREs) (Chen & Shyu, 1995). AREs are determinants of RNA instability in the 3′-UTRs of transcripts and they confer mRNA stabilization by the p38 MAP kinase pathway. A number of proteins have been identified that bind AREs and affect the stability of the transcripts (Dean *et al*., 2004). Thus, the ATTTA motive in the 3′-UTRs of the AmCREB transcripts hints at a regulation of the *AmCREB* gene's expression via the stability of its transcripts.

The number of PAS in E10 was remarkable. It suggests that AmCREB transcripts with variable 3′-ends are generated by alternative polyadenylation. This is supported by the fact that AmCREB-1–4 differed in the length of their 3′-UTRs. The shorter 3′-UTRs contained fewer ATTTA motives than the longer 3′-UTRs (AmCREB-2: five ATTTA, AmCREB-4: 13 ATTTA) and this difference may have an impact on the transcript stability of the different AmCREB variants. A comparable mechanism has been demonstrated for a mammalian member of the CREB/CREM subgroup of transcription factors (Foulkes *et al*., 1993): CREMτ-transcripts are alternatively polyadenylated and the resulting number of ATTTA-motives has an impact on its stability. A reduction in ATTTA-motives using a alternative polyadenylation site leads to an increase of the stability and the amount of this CREMτ-transcript (Foulkes *et al*., 1992, 1993). This mode of regulation may be a common theme in genes of the CREB/CREM subgroup through evolution, because the 3′-UTRs of the *M. musculus CREB* gene (Ruppert *et al*., 1992), the *Drosophila dCREB B* gene (Usui *et al*., 1993) and the *C. virridissima CREB* gene (Galliot *et al*., 1995) contain ATTTA-motives and alternative polyadenylation sites.

## Conclusions

Two genes encoding regulatory subunits and three genes encoding catalytic subunits of PKA were retrieved from the honeybee genome; each resembles a homologue in *Drosophila.* In both species the number of genes encoding a regulatory subunit is half of the corresponding genes in mammals. A reduction of insect catalytic subunits is also observed. The ‘classical’ (three genes in mammals) and the *prkx* (four genes in mammals) subgroups comprise only one gene in insects. Interestingly, it seems that *pka-c2* is specific for insects, as no counterpart was found in humans. A similar reduction of genes holds true for the CREB/CREM family of transcription factors. This has also been found in *Drosophila,* reflecting a reduction from three to one genes in comparison with mammals.

## Experimental procedures

### Searching for new members of PKA and CREB/CREM

#### PKA

BLASTn and BLASTp were used to analyse databases. When searching the honeybee genome (Ensembl v35; http://www.ensembl.org/Apis_mellifera/) with query sequences default parameters were used and the sensitivity of the search was set to ‘near-exact-match’. The results were manually filtered, selecting gene candidates with low *E*-values (< 10e-10) and high scores (> 200). The Blast facility of the BeeBase at the Texas A&M University (http://racerx00.tamu.edu/blast/blast.html) was used to search the ‘official_gene_set_cds_1’ with default parameters.

#### CREB

In tBLASTn and BLASTp searches the ‘official_gene_ set_cds_1’ on the BeeBase, at the Texas A&M University (http://racerx00.tamu.edu/blast/blast.html), was screened with the query sequences. To identify the *Drosophila* and *H. sapiens* homologues, a BLASTp search was carried out with every bZIP sequences identified above, using NCBI Blast (http://www.ncbi.nlm.nih.gov/BLAST/) with default parameters. The retrieved sequences from *Drosophila* and *H. sapiens* proteins with one of the highest scores were chosen and used in the phylogenetic analysis.

### Phylogenetic analysis and comparison of the domains

The deduced amino acid sequences were compared with their *Drosophila* and human homologues by the ClustalW alignment method with the Gonnet series protein weight matrix (default parameters) for the R and C subunits of PKA and with the ClustalV alignment (PAM 250) method using the default parameters for the bZIP proteins, with the MegAlign software (Lasergene, DNAStar, Madison, USA). Unrooted phylogenetic trees, with balanced or unbalanced branches, were generated.

### Gene identification

To identify genes a BLASTn analysis of the official gene sequences against the genome (v2.0) in Ensembl v35 (http://www.ensembl.org/Apis_mellifera/) was done using default parameters.

### AmCREB exon–intron structure

To analyse the exon–intron structure, the different cDNA sequences of the *AmCREB* variants (Eisenhardt *et al*., 2003) were aligned to the identified group using the program Spidey (Wheelan *et al*., 2001). Standard parameters were used, except that ‘genomic sequence’ was set to *Drosophila* and large intron sizes were used. Results were controlled manually.

## Supplementary material

Table S1 Conserved domains within the catalytic subunits

Table S2 Exon-intron structure of the *AmCREB* gene

## References

[b1] Abel T, Bjatt R, Maniatis T (1992). A Drosophila CREB/ATF transcriptional activator binds to both fat body- and liver-specific regulatory elements. Genes Dev.

[b2] Akker SA, Smith PJ, Chew SL (2001). Nuclear post-transcriptional control of gene expression. J Mol Endocrinol.

[b3] Burset M, Seledtsov IA, Solovyev VV (2000). Analysis of canonical and non-canonical splice sites in mammalian genomes. Nucleic Acids Res.

[b4] Canaves JM, Taylor SS (2002). Classification and phylogenetic analysis of the cAMP-dependent protein kinase regulatory subunit family. J Mol Evol.

[b5] Chen CY.A, Shyu AB (1995). AU-rich elements: characterization and importance in mRNA degradation. TIBS.

[b6] Dahle MK, Reinton N, Orstavik S, Tasken KA, Tasken K (2001). Novel alternatively spliced mRNA (1c) of the protein kinase A RI & alpha subunit is implicated in haploid germ cell specific expression. Mol Reprod Dev.

[b7] Dean JL, Sully G, Clark AR, Saklatvala J (2004). The involvement of AU-rich element-binding proteins in p38 mitogen-activated protein kinase pathway-mediated mRNA stabilisation. Cell Signal.

[b8] Eisenhardt D, Fiala A, Braun P, Rosenboom H, Kress H, Ebert PR, Menzel R (2001). Cloning of a catalytic subunit of a cAMP-dependent protein kinase from the honeybee (Apis mellifera) and its localization in the brain. Insect Mol Biol.

[b9] Eisenhardt D, Friedrich A, Stollhoff N, Müller U, Kress H, Menzel R (2003). The AmCREB gene is an ortholog of the mammalian CREB/CREM family of transcription factors and encodes several splice variants in the honeybee brain. Insect Mol Biol.

[b10] Fassler J, Landsman D, Acharya A, Moll JR, Bonovich M, Vinson C (2002). B-ZIP proteins encoded by the Drosophila genome: evaluation of potential dimerization partners. Genome Res.

[b11] Fiala A, Müller U, Menzel R (1999). Reversible downregulation of protein kinase A during olfactory learning using antisense technique impairs long-term memory formation in the honeybee, Apis mellifera. J Neurosci.

[b12] Foulkes NS, Mellström B, Benusiglio E, Sassone-Corsi P (1992). Developmental switch of CREM function during spermatogenesis: from antagonist to activator. Nature.

[b13] Foulkes NS, Schlotter F, Pévet P, Sassone-Corsi P (1993). Pituitary hormone FSH directs the CREM functional switch during spermatogenesis. Nature.

[b14] Francis SH, Corbin JD (1999). Cyclic nucleotide-dependent protein kinases: Intracellular receptors for cAMP and cGMP. Crit Rev Clin Lab Sci.

[b15] Friedrich A, Thomas U, Muller U (2004). Learning at different satiation levels reveals parallel functions for the cAMP-protein kinase A cascade in formation of long-term memory. J Neurosci.

[b16] Galliot B, Welschof M, Schuckert O, Hoffmeister S, Schaller HC (1995). The cAMP response element binding protein is involved in hydra regeneration. Development.

[b17] Geballe AP, Hershey JWB, Mathews MB, Sonenberg N (1996). Translational control mediated by upstream AUG codons. Translational Control.

[b18] Gonzalez GA, Menzel P, Leonard J, Fischer WH, Montminy MR (1991). Characterization of motives which are critical for activity of the cyclic AMP-responsive transcription factor CREB. Mol Cell Biol.

[b19] Guthrie CR, Skalhegg BS, McKnight GS (1997). Two novel brain-specific splice variants of the murine Cbeta gene of cAMP-dependent protein kinase. J Biol Chem.

[b20] Hai T, Hartman MG (2001). The molecular biology and nomenclature of the activating transcription factor/cAMP responsive element binding family of transcription factors: activating transcription factor proteins and homeostasis. Gene.

[b21] Kalderon D, Rubin GM (1988). Isolation and characterization of Drosophila cAMP-dependent protein kinase genes. Genes Dev.

[b22] Kandel ER (2001). The molecular biology of memory storage: a dialogue between genes and synapses. Science.

[b23] Knighton DR, Zheng JH, Ten Eyck LF, Ashford VA, Xuong NH, Taylor SS, Sowadski JM (1991). Crystal structure of the catalytic subunit of cyclic adenosine monophosphate-dependent protein kinase. Science.

[b24] Kozak M (2002). Pushing the limits of the scanning mechanism for initiation of translation. Gene.

[b1a] Laoide BM, Foulkes NS, Schlotter F, Sassone-Corsi P (1993). The functional versatility of CREM is determined by its modular structure. EMBO J.

[b25] Leboulle G, Muller U (2004). Synergistic activation of insect cAMP-dependent protein kinase A (type II) by cyclic AMP and cyclic GMP. FEBS Lett.

[b26] Li X, Li HP, Amsler K, Hyink D, Wilson PD, Burrow CR (2002). PRKX, a phylogenetically and functionally distinct cAMP-dependent protein kinase, activates renal epithelial cell migration and morphogenesis. Proc Natl Acad Sci USA.

[b27] Liu F, Thompson MA, Wagner S, Greenberg ME, Green MR (1993). Activating transcription factor-1 can mediate Ca(2+)- and cAMP-inducible transcriptional activation. J Biol Chem.

[b28] Lonze BE, Ginty DD (2002). Function and regulation of CREB family transcription factors in the nervous system. Neuron.

[b29] Masquilier D, Foulkes NS, Mattei MG, Sassone-Corsi P (1993). Human CREM gene: evolutionary conservation, chromosomal localization, and inducibility of the transcript. Cell Growth Differ.

[b30] Meijer HA, Thomas AA (2002). Control of eukaryotic protein synthesis by upstream open reading frames in the 5′-untranslated region of an mRNA. Biochem J.

[b31] Melendez A, Li W, Kalderon D (1995). Activity, expression and function of a second Drosophila protein kinase A catalytic subunit gene. Genetics.

[b32] Menzel R (2001). Searching for the memory trace in a mini-brain, the honeybee. Learn Mem.

[b33] Orstavik S, Reinton N, Frengen E, Langeland BT, Jahnsen T, Skalhegg BS (2001). Identification of novel splice variants of the human catalytic subunit Cbeta of cAMP-dependent protein kinase. Eur J Biochem.

[b34] Orstavik S, Funderud A, Hafte TT, Eikvar S, Jahnsen T, Skalhegg BS (2005). Identification and characterization of novel PKA holoenzymes in human T lymphocytes. FEBS J.

[b35] Poels J, Vanden Broeck J (2004). Insect basic leucine zipper proteins and their role in cyclic AMP-dependent regulation of gene expression. Int Rev Cytol.

[b36] Quinn PG (1993). Distinct activation domains within cAMP response element-binding protein (CREB) basal and cAMP-stimulated transcription. J Biol Chem.

[b37] Ruppert S, Cole TJ, Boshart M, Schmid E, Schuetz G (1992). Multiple mRNA isoforms of the transcription activator protein CREB: generation by alternative splicing and specific expression in primary spermatocytes. EMBO J.

[b38] Schiebel K, Mertz A, Winkelmann M, Glaser B, Schempp W, Rappold G (1997). FISH localization of the human Y-homolog of protein kinase PRKX (PRKY) to Yp11.2 and two pseudogenes to 15q26 and Xq12→q13. Cytogenet Cell Genet.

[b39] Short S, Manohar CF, Furtado MR, Ghadge GD, Wolinsky SM, Thimmapaya B, Jungmann RA (1991). Nucleotide and derived amino-acid sequences of CRE-binding proteins from rat C6 glioma and HeLa cells. Nucleic Acids Res.

[b40] Smolik SM, Rose RE, Goodman RH (1992). A cyclic AMP-responsive element-binding transcriptional activator in D. melanogaster, dCREB-A, is a member of the leucine zipper family. Mol Cell Biol.

[b41] Tasken K, Aandahl EM (2004). Localized effects of cAMP mediated by distinct routes of protein kinase A. Physiol Rev.

[b42] Taylor SS, Kim C, Vigil D, Haste NM, Yang J, Wu J, Anand GS (2005). Dynamics of signaling by PKA. Biochim Biophys Acta.

[b43] Usui T, Smolik SM, Goodman RH (1993). Isolation of Drosophila CREB-B: a novel CRE-binding protein. DNA Cell Biol.

[b44] Wahle E, Keller W (1992). The biochemistry of 3′-end cleavage and polyadenylation of messenger RNA precursors. Annu Rev Biochem.

[b45] Wheelan SJ, Church DM, Ostell JM (2001). Spidey: a tool for mRNA-to-genomic alignments. Genome Res.

[b46] Wu Q, Krainer AR (1999). AT-AC pre-mRNA splicing mechanisms and conservation of minor introns in voltage-gated ion channel genes. Mol Cell Bio.

[b47] Yin JC, Wallach JS, Wilder EL, Klingensmith J, Dang D, Perrimon N (1995). A Drosophila CREB/CREM homolog encodes multiple isoforms, including a cyclic AMP-dependent protein kinase-responsive transcriptional activator and antagonist. Mol Cell Biol.

[b48] Zimmermann B, Chiorini JA, Ma Y, Kotin RM, Herberg FW (1999). PrKX is a novel catalytic subunit of the cAMP-dependent protein kinase regulated by the regulatory subunit type I. J Biol Chem.

