# Supplementary figures and images for: The PKA-CREB system encoded by the honeybee genome
Source: Insect Mol Biol. 2006 Oct;15(5):551–61. doi: 10.1111/j.1365-2583.2006.00668.x (PMC1769443; doi:10.1111/j.1365-2583.2006.00668.x)

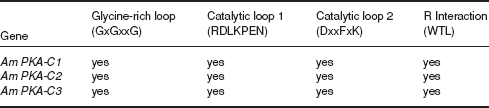

Supplement: Table S1 — Conserved domains within the catalytic subunits [file imb0015-0551-s1.tif]

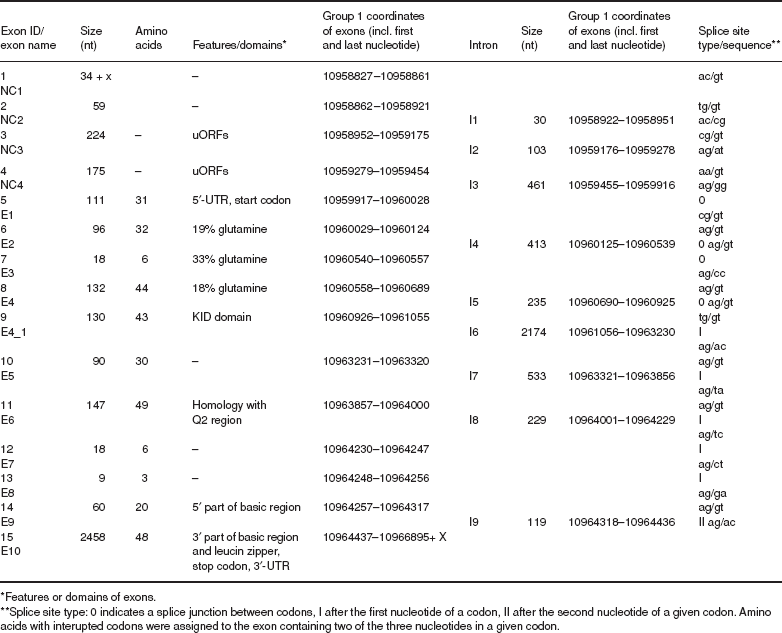

Supplement: Table S2 — Exon-intron structure of the AmCREB gene [file imb0015-0551-s2.tif]
